# Health Tract, No. 302

**Published:** 1867-08

**Authors:** 


HEALTH TRACT, No. 302.

### AMATEUR PHYSICIANS.

If a layman is recommended by any one to take or do something for an ailment and it is promptly followed by the removal of the thing complained of, he forthwith, from that single instance, becomes enthusiastic, and the very next time he meets with one who has similar "symptoms," he prescribes with great confidence and if that is also successful, he in a very short time will be found giving the same prescription for every thing, it at once becomes in his estimation a panecea, a universal remedy, a cure for everything. It would require scores of such successes and a whole year's, or even five years' observation, for an experienced physician to have a hundredth part of the confidence in any remedy, simply because he knows the uncertainties of remedies, and how rare it is that the same conditions are found in two cases. The use of remedies for the alleviation and cure of ailments was very simple in the earlier ages of the world. Then men were inclined for purposes of self-defence to live close together, and to surround the place with high stone walls with gates at convenient distances; these places were called cities. The gates were opened in the morning and closed at night, and all persons visiting the city or leaving it, had to pass through these, hence the sick "sat in the gate," in the hope that some one of the multitudes passing might have suffered in the same way, or have seen some one thus suffering, and having been cured would be willing, as an act of humanity, to tell what was done. The older a person was the more he had seen and the more knowledge would he have on the subject; it was a natural and easy step for these old persons to have the remedies prepared and then to apply them, as they had seen them applied; the remedies cost something, and it took time to apply them, hence the idea of compensation for time and trouble arose, and thus it became a regular calling, and thus were made the first doctors, and perhaps these were better than many of our later day, because they practiced from actual observation, while it is common in our time to get the knowledge from books—a much more uncertain method. The older, the safer, is a good general rule; and a better one still is, pay no attention to what outsiders may say about health and disease, however eminent in their calling. Health publications written for by political hacks, clerical sensationaists and lay lecturers are very unsafe guides. One of these has lately had the frankness to acknowledge, "I wrote an article on health some years ago for the ——, and the editors were foolish enough to publish my article," which, he goes on to confess, had no sense in it. We only say that if his "Divinity" gives as uncertain a light as his physic, he had a great deal better go to cracking stones for the turnpike; and the real truth is, some of his 'brethren' have intimated as much. We are very certain old John Knox would, any how.

